# Challenges and Innovations in Pharmacovigilance and Signal Management During the COVID-19 Pandemic: An Industry Perspective

**DOI:** 10.3390/vaccines13050481

**Published:** 2025-04-29

**Authors:** Maria Maddalena Lino, Susan Mather, Marianna Trani, Yan Chen, Patrick Caubel, Barbara De Bernardi

**Affiliations:** 1 Vaccine Research and Development and Worldwide Safety, Safety Surveillance and Risk Management, Pfizer, 20152 Milan, Italy; marianna.trani@pfizer.com (M.T.); barbara.debernardi@pfizer.com (B.D.B.); 2 Vaccine Research and Development and Worldwide Safety, Safety Surveillance and Risk Management, Pfizer, Collegeville, PA 10965, USA; susanmather215@gmail.com (S.M.); yan.chen7@pfizer.com (Y.C.); 3 Vaccine Research and Development, Pfizer, Pearl River, New York, NY 19426, USA

**Keywords:** signal detection, signal evaluation, COVID-19 vaccine, efficacy, safety, adverse event following immunization, COVID-19 variants, pandemic

## Abstract

Vaccine marketing authorization holders (MAHs) are responsible for the conduction of global vaccine pharmacovigilance on their vaccine products. A safety signal is detected when a new adverse event (AE) or aspect of an AE occurs after exposure to the vaccine and warrants further investigation to determine whether a causal association may exist. Signal detection and evaluation (signal management) begins at the start of vaccine development, before an MAH submits an application for authorization to regulatory authorities, continues through the course of all clinical trials, and carries on beyond development into the post-marketing phase. As long as the vaccine remains authorized anywhere in the world, pharmacovigilance continues. During the time that the COVID-19 vaccine became widely available after authorization and approval, clinical trials were also ongoing, and therefore all clinical development and post-authorization safety information was closely monitored for safety by the MAH. MAH pharmacovigilance activities were adapted to manage the unprecedented volume of safety information that became available within a very short timeframe following worldwide vaccination campaigns. No vaccine had previously been administered to such a large number of individuals in such a short time, nor had there previously been a public health vaccine experience that was the subject of so many medical and non-medical writings. The MAH’s COVID-19 vaccine signal detection methods included the continuous review of accruing clinical trial data and the quantitative and qualitative analyses of spontaneously reported experiences. Review of published and unpublished medical literature and epidemiology-based analyses such as observed vs. expected analysis based on reported adverse events following immunization (AEFIs) played key roles in pharmacovigilance and signal management. All methods of signal detection and evaluation have caveats, but when considered in totality, can advance our understanding of a vaccine’s safety profile and therefore the risk–benefit considerations for vaccinating both individuals and large populations of people. All COVID-19 vaccines authorized for use were subject to an unprecedented level of pharmacovigilance by their individual MAHs, national regulatory authorities, public health organizations, and others during the years immediately following regulatory authorization and full approval. The intense worldwide focus on pharmacovigilance and the need for MAHs and regulatory/health authorities to quickly evaluate incoming safety information, spurred frequent and timely communications between national and regional health authorities and between MAHs and regulatory/health authorities, spotlighting a unique opportunity for individuals committed to patient safety to share important accruing safety information in a collegial and less traditionally formal manner than usual. The global pandemic precipitated by the SARS-CoV-2 virus created a significant impetus for MAHs to develop innovative vaccines to change the course of the COVID-19 pandemic. Pharmacovigilance also had to meet unprecedented needs. In this article, unique aspects of COVID-19 vaccine pharmacovigilance encountered by one MAH will be summarized.

## 1. Introduction

### Pre COVID-19 Pandemic Vaccine Pharmacovigilance

Quantitative and qualitative analyses of safety data collected for a new vaccine are the basis of post-authorization signal detection. It is obligatory to good pharmacovigilance practice (GVP) to conduct appropriate quantitative disproportionality analyses [1] on a regular basis for safety signal detection (good pharmacovigilance practices (GVPs) VIII, IX). Disproportionality analyses of spontaneously reported AEFIs tend to be sensitive in picking up potential signals, but this can vary depending on the nature of the database and on the parameters used. For example, some safety reporting databases may contain only vaccine AE reports (e.g., VAERS), whereas MAH safety databases generally contain AE reports for all of the company’s medicinal products, vaccine and non-vaccine. Additionally, parameters regarding the disproportionality ratio that stimulates further investigation should be established based on prevailing standards. Regardless, findings should always be interpreted in the context of the totality of available safety data, including qualitative analyses. The qualitative analysis of spontaneously reported AEFIs requires consideration of the clinical details in each case, including those supporting the accuracy of the reported AEFI, the timing between vaccination and the AEFI, and any contextual factors that may be consequential or confounding to the case. In addition, the safety data amassed from the vaccine clinical trials cannot be discounted. Even though it is usually collected from a relatively small, homogeneous, and medically stable population of participants, its controlled nature provides clarity of interpretation. Large pivotal clinical trials with thousands of participants are generally too small to detect the occurrence of infrequent or rare adverse reactions; however, these controlled trials are critical for initial signal detection and form the basis for the known safety profile (i.e., adverse reactions) expected of the vaccine at the time of authorization [2].

This article provides an industry perspective on the conduction of post-authorization safety surveillance for the first authorized COVID-19 vaccine, highlighting unique challenges to pharmacovigilance due to the global pandemic environment.

## 2. Relevant Sections

### 2.1. COVID-19 Pandemic Pharmacovigilance Challenges and Opportunities

The COVID-19 pandemic declared by the World Health Organization (WHO) on 11 March 2020, resulted in a health crisis with a devastating and global impact on human lives and national economies. The exceptional feat of development and emergency authorization of the COVID-19 vaccines during the ongoing pandemic changed its course. Indeed, COVID-19 vaccines have been highly effective at protecting individuals, especially medically vulnerable individuals, against severe COVID-19 including hospitalizations, and mortality. While the ability of the SARS-CoV-2 virus to rapidly mutate hinders the elimination of the virus from circulation, successful vaccination programs have allowed people to weather the pandemic with decreased morbidity and mortality and to resume most regular activities.

#### 2.1.1. Rapid and Large-Scale Worldwide Vaccination Campaigns

Marketing authorization holders and those conducting vaccine pharmacovigilance on their behalf, prepared for the large volumes of AEFI reports expected once the vaccines were authorized, distributed and administered in large vaccination campaigns, but the unprecedented number of individual case safety reports (ICSRs) received within a short time exceeded expectations based on the previous H1N1 pandemic. The MAH safety database infrastructure to process the ICSRs in accordance with regulatory timelines required additional and continual reinforcement long after the initial vaccination campaigns. Regulatory authorities the world over also faced the burden of overwhelming numbers of ICSRs within a short time. Mitigation against a lag in assessment of important safety data in these circumstances required prioritizing the processing of serious cases ahead of non-serious cases. It took some regulatory authorities longer than others to complete the processing of their backlog of cases for dispersion to MAHs. The boluses of cases received by MAHs, therefore, did not necessarily indicate a new pattern of AEFIs being experienced; rather, they often indicated that a regulatory authority was “catching up” with their ICSR processing. Under ideal circumstances, all ICSRs, regardless of seriousness, are individually processed and assessed in real-time, reflecting the real-time experience of vaccinees. The rapid influx and, at times, the ebb and flow of ICSRs received from regulatory authorities made this impossible in the case of COVID-19 vaccines. Knowledge of local regulatory authority activities and awareness of this occurrence helped the MAH be prepared and interpret the flow of vaccination experiences accurately. This was a key lesson that could aid pharmacovigilance efforts if similar situations occur in the future.

A unique and valuable consequence of rapid large-scale COVID-19 vaccination campaigns to early post-authorization MAH pharmacovigilance was the purposefully close communication and exchange of post-vaccination safety information that occurred in routine and ad hoc meetings between regulatory/health authorities and MAHs. This close collaboration allowed for rapid sharing of safety data trends and the discussion of safety assessments and issues, as safety information rapidly accrued.

#### 2.1.2. Prioritizing Vaccination of High-Risk Populations

Following vaccine authorizations and approvals, national regulatory and health authorities prioritized the distribution and administration of the COVID-19 vaccines according to their country’s needs. In general, vaccinations were administered in a staggered fashion, prioritizing vaccination in patients and individuals with increased risk of severe COVID-19 and death (e.g., elderly individuals in long-term care facilities), followed by healthcare professionals and essential workers [3]. This phasic pattern of vaccine administration influenced the nature of AEFI reporting such that many initial ICSRs regarded medically vulnerable individuals. While it is logical to expect that elderly and medically vulnerable individuals with multiple comorbidities and on concomitant medications are more likely to experience serious medical events regardless of vaccine exposure, it must also be considered that such individuals may be more susceptible to AEs potentially precipitated by vaccination. Early AEFIs reported from the first vaccinated individuals in the post-authorization period were at times different and more serious and/or severe than the AEFIs reported in the early-vaccinated healthcare professionals and the clinical trial participants which consisted largely of mild to moderate reactogenicity events (e.g., fever, muscle and joint aches, headache) typical of vaccination. Nevertheless, as the administration, collection and assessment of data by the MAH and regulatory authorities progressed (through primary and booster dosing), the favorable benefit/risk profile of the COVID-19 vaccine in elderly and medically vulnerable populations remained clear. Continuously monitoring and analysing the AEFIs by populations (e.g., pediatrics, elderly and patients with underlying comorbidities) was critical to ensure the safety in those vulnerable populations.

It is worth emphasizing that real-world data are not as easily interpreted as clinical trial data. Normal life circumstances introduce countless factors that impact individual health and wellness. Untangling and understanding the contextual factors influencing AE reporting takes time but is necessary in order to soundly assess a vaccine safety profile. It is expected that a product’s safety profile will evolve as post-authorization use progresses beyond the populations studied in the pivotal clinical trials; however, the rapid discernment of whether reported AEs are coincidental to vaccination or actually caused or made worse by the vaccine can be very challenging. During the height of COVID-19 vaccination, time was of the essence. Circumstances were more challenging than usual due to the well-intentioned global efforts to report all AEFIs and due to the exceptionally close media scrutiny on any and all information about the safety and effectiveness of the pre-licensed authorized vaccines, particularly those utilizing the novel mRNA platform. These circumstances, along with the mandate to vaccinate in many regions, led some individuals and groups to regard scientific information from MAHs and health authorities with wariness. Indeed, there was no shortage of available opinions on vaccination risks and events that were possibly but unlikely to be related to vaccination. Opinions by vaccine experts and non-experts were freely shared and debated on the internet and television and were not always easy for lay people to decipher. Information about COVID-19 vaccine safety from reliable sources such as product labeling and national health authorities was also available however it competed for public attention with information that was disparate, incomplete, or completely lacked a basis in data-driven assessments of reliable data. This climate of information overload, particularly online information lacking validity and reliability, very likely led to confusion among many. And because talk of COVID-19 and the vaccines dominated many online forums, it quite possibly influenced AEFI-reporting behavior. At the least, the volume of safety information collected, databased, and assessed was a challenge beyond previous new-product pharmacovigilance experience.

Overall, the post-authorization safety profile of the Pfizer/BioNTech COVID-19 vaccine remained similar to that observed in clinical trials in all ages and populations.

#### 2.1.3. Regional Differences in Pharmacovigilance and AEFI Reporting

The manner and magnitude of pharmacovigilance conducted by national regulatory authorities affects the regional safety information that reaches the MAH for assessment. Many countries with institutionally supported and established pharmacovigilance infrastructures and many in-country research institutions collected post-vaccination follow-up information including AEFIs from groups of vaccinees. While efforts to collect and study vaccine safety and effectiveness data are critical and commendable, the quality of safety data from national authorities and researchers varied.

Similarly, the flow and communication of collected data resulted in challenges for the MAH conduction of review and assessment of safety data globally. Regional patterns of AEFI-reporting to the MAH were observed. For example, a country with approximately 10 million inhabitants reported as many AEFIs as countries with 70, 90, and even more than 300 million inhabitants. Efforts made to collect post-vaccination data differed among countries and also, seemingly, in terms of types of AEs (e.g., serious, non-serious) and populations targeted (e.g., care homes, healthcare workers). These differences along with the aforementioned boluses of ICSRs from health authorities are understandable but challenging, as it was not possible to know the planned pharmacovigilance initiatives of all countries. Some regulatory authorities who collected or received overwhelming numbers of ICSRs understandably had to prioritize processing and forwarding of those ICSRs to MAHs.

A challenge in MAH pharmacovigilance that seemed accentuated due to the high volume of ICSRs was inconsistency in MedDRA coding (e.g., choice of preferred term [PT]), seriousness assessment, and the amount of clinical detail in the reports. An example of previously observed regional differences in coding is that some regions may use the PT *hyperpyrexia* (with no provision of numerical temperature data) rather than *fever*. Indeed, it is not unexpected that varying country resources will impact ICSR quality and that regional conventions may lead to idiosyncrasies in coding; however, a large volume of ICSRs seems to exacerbate those differences. It is also important to know that MAH conventions generally prohibit changes to ICSR coding received from regulatory authority sources. A notable example of specific regional AEFI reporting was reports of menstruation abnormalities. These reports were disproportionately reported from European countries compared to the US and an explanation for such regionality is not completely understood.

An apriori understanding of national health authority plans for post-vaccination safety data acquisition in their regions would be of future value to MAHs, who must consider data from each country and globally in their worldwide pharmacovigilance responsibilities. While much has been made of the benefits of international regulatory authority co-operation, not enough has been said about the potential benefits of enhanced communication by regulatory authorities to the MAHs about country and regional pharmacovigilance initiatives. MAHs are beholden by country regulations and prevailing international standards to keep regulatory authorities informed of product safety information. Improvements in efficiency and responsible resource stewardship could be gained by all if regulatory/health authorities informed MAHs of their PV plans beyond routine activities. Continued open communication between MAHs and regulatory/health authorities beyond the initial months after vaccine authorization would provide valuable contextualization of country-specific AE reports sent to the MAH.

#### 2.1.4. Differences in Primary Vaccination Intervals and Primary vs. Booster Vaccinations

In each MAH COVID-19 vaccine clinical trial, the interval between the two primary vaccinations was clearly defined. For the pivotal the Pfizer/BioNTech COVID-19 vaccine trials, the interval was 21 days. However, in real-world vaccination, some countries altered the interval based on their individual country goals and needs as well as their supply of vaccines. Some countries prioritized attaining partial COVID-19 protection for a large number of individuals over complete protection for a smaller number of individuals. Obtaining a degree of protection for the majority in the shortest period of time is a benefit–risk consideration that is the prerogative of each country and one manner in which the preservation of the healthcare system may be ensured. The interval between primary vaccination doses went to 42 days or more in some cases (up to 8–12 weeks in the UK) [4]. As well as expanding the recommended 21-day interval between the first and second (primary) vaccinations, some countries also elongated the interval between the second and third (booster) vaccination compared to the interval studied in clinical trials. As the world was learning about SARS-CoV-2’s response, vaccination intervals that had not been studied surely impacted AEFI reporting practices, including reports of the lack of efficacy which were liberally reported even when vaccination interval recommendations (based on MAH clinical trials) were not followed. As AEFI reporting was impacted, so too was MAH ICSR processing and signal management.

#### 2.1.5. Heterologous COVID-19 Vaccinations

Just as some countries needed to adjust dosing intervals based on vaccine supply and availability, the exceptional circumstances of the pandemic paved the way for health authorities to allow heterologous vaccination. Heterologous vaccination meant that an individual was not required to be vaccinated with the same manufacturer’s vaccine for all of their administered doses. Indeed, most health authorities supported individuals receiving different brands of vaccine and in many cases, different vaccine platforms (e.g., viral vector, mRNA, and antigen-based) were also interchanged [5]. In time, observational study findings were supportive of heterologous vaccination.

As with multiple vaccination doses, the capture of information within AEFI reports became more complicated as varying vaccination possibilities for each dose (e.g., three different vaccine manufacturers for dose 1, dose 2, and dose 3) progressed. Such product and administration detail, while critical for surveillance, was an added factor in case processing that could lead to errors or missing information when reported by vaccinees and transcribed in AE reports. The sheer complexities of accurately documenting multiple doses or multiple brand of vaccines administered over differing intervals made the ICSRs more complex to review and understand.

While the practice of heterologous vaccination may be presumed to have little impact on the capture of acute AEFIs such as reactogenicity events, it could theoretically complicate the appreciation of unknown potential risks of vaccination that may be cumulative or may manifest long after the time of first vaccination, especially given the lack of studies for all dosing and regimen permutations.

Eventually, the efficacy and safety of heterologous vaccination was judged to be adequate and acceptable as research suggested that it triggered cellular and humoral responses comparable to those observed with homologous vaccination and washighly effective in preventing severe COVID-19 disease with no major safety concerns observed [6,7,8,9,10,11]. Some studies even showed that heterologous vaccination elicited higher immunogenicity than homologous vaccination which may be predictive of better efficacy [12,13,14,15]. Overall, both homologous and heterologous booster vaccines regimens have been shown to be safe and effective in adults who had completed a primary COVID-19 vaccine regimen at least 12 weeks earlier [16,17].

#### 2.1.6. Circulation of Different COVID-19 Variants

When the initial COVID-19 vaccines were authorized, the durability of vaccine effectiveness beyond a few months was unstudied. SARS-CoV-2 variants due to rapidly occurring spike mutations continued to emerge and produce variants of concern (VOCs) and variants of interest for which vaccines were needed since robust immunity against the virus from previous wild-type infection or vaccination was not long-lived. This changing virus epidemiology also contributed to the challenges of continuous vaccine pharmacovigilance [18]. Chronologically, the emergence of the beta, delta, and omicron VOCs was associated with new waves of worldwide infection, the most serious of which seemed to occur in unvaccinated individuals; individuals vaccinated against previous strains also experienced immune evasion and infection [19,20]. The transmissibility of SARS-CoV-2 increased dramatically with the omicron VOC, which was first identified in South Africa. In a short time, omicron overtook delta as the dominant variant around the world. The first iteration of mRNA COVID-19 vaccines had been highly effective against the alpha variant, and less effective against beta, gamma, and delta variants, thus making it necessary to develop adapted mRNA vaccines [21]. As time passed and Omicron variants emerged, the mRNA vaccines were updated to maintain vaccine effectiveness [22]. The new variant vaccines were authorized by regulatory authorities and made available to increase neutralizing antibody titres and effectiveness against the new variants.

With each updated vaccine, fewer doses have been administered. Although the virus continues to circulate, causing severe disease and death, the incidence of severe disease, hospitalizations and deaths has decreased overall due to the immunity afforded by vaccination and infection, and, possibly, due to decreased virus virulence. Many health authorities have discontinued tracking COVID-19 cases, hospitalizations and deaths, and, therefore, accurate estimations of vaccine effectiveness and the conduction of certain pharmacovigilance efforts (e.g., observed to expected analyses) have become more challenging due to lack of exposure data. Also challenging is that worldwide reports of AEFIs are now reported by individuals with mixed vaccine experiences, for example: no previous COVID-19 vaccinations, heterologous vaccination, homologous vaccination, and partial COVID-19 vaccination. The diversity of vaccine experiences can complicate the assessment of AEs with respect to the most recently administered updated vaccine. As one example, reports of lack of efficacy (LOE) may not be accurate if a reporter with COVID-19 is not up to date with their vaccinations. At this time, and likely due to the unchanged formulation of the vaccine (except for the spike protein portion), the safety profile of each updated vaccine, as studied in clinical trials and assessed in the post-marketing setting, have remained very similar to the original mRNA COVID-19 vaccine.

#### 2.1.7. Lack of Standardization in AEFI Reporting and Potential ICSR Duplication

Regional differences in how AEFIs are coded is not a new phenomenon in pharmacovigilance. In some countries, regulatory authorities and/or healthcare professionals tend to use specific preferred terms (PTs) or conventions to describe AEFIs. The use of a single medical dictionary, MedDRA, for coding events in ICSRs is of great value in AEFI reporting. MedDRA’s hierarchy and organization, including SMQs, helps to organize coding variations. Even with MedDRA, however, there are still inconsistencies and differences in the use of terminology in AE reporting that may at times necessitate changes in safety database search strategies to ensure focus on appropriate cases. The search strategies of safety databases should be appropriately wide (sensitive) or narrow (specific) depending on the signal detection or evaluation needs. As adverse events reported coincident with COVID-19 vaccination quickly outnumbered all other product AEs within safety databases, the ability to perform appropriate searches became even more critical.

A particular challenge when assessing spontaneous reports is that even when reported by healthcare professionals or regulatory authorities, events coded may inconsistently provide detail or lack details to support a proper assessment of the case. When assessing some adverse events of specific interest (AESIs), it is helpful to have standardized criteria to assess the likely diagnostic accuracy of the event reported. Case definitions and companion guides developed for a number of AESIs by the Brighton Collaboration, an independent vaccine expert group supported by the global Coalition for Epidemic Preparedness Innovations (CEPI), were useful when available. The use of these tools enabled the placement of ICSRs of specifically reported events into a hierarchy of certainty regarding the occurrence of the AESI, allowing focus on the most informative ICSRs. Using Brighton Collaboration tools also provided unbiased methodology, due to their independent expert status, and therefore, a measure of reassurance in the objective assessment of safety data.

Additionally, the Brighton Collaboration and Safety Platform for Emergency Vaccines (SPEAC) produced a list of conditions that they considered COVID-19 vaccine AESIs. Many regulatory and health authorities also used lists of AESIs in their COVID-19 safety surveillance and pharmacovigilance activities. Early after authorization of the first COVID-19 vaccines, the variability in regulatory authority communication and expectations regarding the use of specific AESI lists for MAH pharmacovigilance was challenging. The availability of multiple AESI lists that were similar but not identical was another example where harmonization would improve efficiency. The operationalization of AESI lists using the MedDRA dictionary remains another aspect of pharmacovigilance that may be beneficial to standardize. 

Another challenge when conducting surveillance using post marketing reports is the potential duplication of reported cases. This can occur when different sources report on the same case and the system fails to link follow-up reports to original records. Duplicate reporting has always been a consideration with spontaneous reporting; for example in the publication by Manfred et al. [23], a case of extreme duplication was found in the US FDA Adverse Events Reporting System (AERS) database. During a data-mining demonstration, it was discovered that most reports of a drug–event pair were duplicates. This could potentially lead to a misleading signal of disproportionate reporting. The example underscores the importance of thorough data cleaning procedures.

#### 2.1.8. Use of Observed to Expected Analyses in Signal Detection

As a pharmacovigilance tool, observed to expected (O/E) analyses have generally been used after a safety signal has been raised from another source (e.g., disproportionate reporting, literature reviews) to aid in signal refinement and assessment [24]. However, O/E analyses for signal detection were used in COVID-19 vaccine safety surveillance largely due to the unprecedented volume of ICSRs received. Indeed, EMA regulatory pharmacovigilance expectations included O/E analyses for AESI signal detection [25]. O/E analyses served as a tool to seek disproportionality in the occurrence of some AESIs.

In many instances, the quality of individual post-marketing ICSRs render a clinical evaluation of the diagnosis and potential causal role of the vaccine difficult. Aggregating the reported ICSRs (as the observed portion or numerator) and using an established background rate (as the expected portion or denominator) allows the conduction of O/E analysis for signal detection in a spontaneous database. A conservative approach may be taken for signal detection by including all the reports of an event regardless of diagnostic certainty. When ICSRs contain adequate data and background rates differ by population but are well established, O/E analyses may be stratified by age, sex, doses, calendar time, and other relevant factors. Hence, O/E analysis became a routine aspect of both signal detection and refinements when appropriate and as long as input data were sufficiently available. Regarding background rates, the information provided by ACCESS was a useful tool for some AESIs; however, for other AESIs, it became apparent that more up to date and population-specific background rates were needed. The continual surveillance and updating of background rates of key AESIs would help preparedness for similar surveillance needs in the future. Updates are especially needed for those events that may be more easily diagnosed due to medical advances than when previous background rates were published.

There are known limitations associated with O/E analysis. For example, the analyses are often contingent on a few assumptions which, when violated, may lead to biased estimates. Nevertheless, O/E analysis is a useful tool for strengthening and refining signal detection, particularly when rapid conclusions about the safety of a vaccine are needed and the events of interest are acute.

#### 2.1.9. Pre-Published Reports and Medical Publications

While literature analysis has always been a valuable aspect of signal detection and evaluation, the amount of vaccine safety literature produced after the authorization of the COVID-19 vaccines was unprecedented. A rush to publish important safety information for the benefit of individual and public health is critical. In the case of the COVID-19 vaccines, the sheer amount of information seemingly led to publications of variable quality. The resulting overabundance of information required a small army to keep abreast of the literature and to separate the wheat from the chafe.

Disregarding the availability of information of questionable scientific quality that did not go through an adequate peer or other review process, there were innumerable cases in which the published literature was very valuable in further understanding safety signals detected. Examples included the assessment of post-vaccination flares or the worsening of underlying conditions in individuals with various pre-existing comorbidities. It is difficult to assess a worsening/flare of a disease without knowing all aspects of the disease in the subject as each person can have their own “flare incidence rate” that may depend on different aspects such as concomitant infection, stressful situation, change of medications, etc. In some cases, to improve the mounting of antibody responses after vaccination, physicians may reduce the underlying immunosuppressant treatments in patients with autoimmune diseases. This alone may contribute to a flare of the underlying disease contemporaneous with vaccination. Informative studies were published by specialized disease centers reporting disease-specific experiences in panels of patients after COVID-19 vaccination. When the specialized center had a full overview of their patients including medical status and concomitant medications assessments could be made with minimal bias. Such publications supported the surveillance and monitoring of COVID-19 vaccine safety profiles in potentially vulnerable patient populations.

### 2.2. A Unique Experience with One Country

The Pfizer/BioNTech COVID-19 vaccine was initially the sole COVID-19 vaccine supplied to the country of Israel. This allowed a unique opportunity for safety and effectiveness observations during the earliest vaccination campaigns. The Israeli Ministry of Health was very communicative with Pfizer/BioNTech and shared information about their experience with the vaccine, including adverse events reported to them following vaccinations. This, along with their safety queries to Pfizer/BioNTech, promoted open collaboration which allowed the reassurance of the vaccine safety profile observed in the clinical trials, while also being a source of new important safety information. This information-sharing was a model for emulation in early post-authorization regulatory and industry relations.

## 3. Conclusions and Future Directions

For the first time in history, the world was able to benefit in real-time from a vaccine developed during a deadly pandemic. Effective vaccines were rapidly conceived, studied, manufactured at scale, authorized, and administered worldwide due to the combined efforts of thousands of people within industry, healthcare and government working in concert globally.

The resulting pharmacovigilance experiences shared highlight some of the unique and challenging aspects of conducting safety surveillance on a new vaccine product in the context of the global pandemic. The foundational activities of pharmacovigilance occurred and adaptations were made to handle the unprecedented volume of safety information resulting from multiple and continuous large global vaccination campaigns. New applications for O/E analyses were utilized and more open communication between industry and regulatory authorities was experienced.

Our experiences provide insight into opportunities for improvements in pandemic pharmacovigilance readiness. While regulatory/health authorities and MAHs made their strong commitment to safety surveillance and pharmacovigilance evident by their collaborative work and timely dissemination of important safety information, further improvements in preparedness can be made by heeding the lessons of the COVID-19 pandemic. Collaboration, open communication regarding regional vaccination campaign plans and national pharmacovigilance plans, and as much standardization in the collection of post-authorization safety information as possible will improve our safety surveillance of vaccines in future global public health emergencies.
